# Quorum sensing in the *Burkholderia cepacia* complex: biosynthesis, functions, and signaling pathways

**DOI:** 10.1128/aem.02020-25

**Published:** 2026-01-06

**Authors:** Xiaohan Kong, Xiaohui Li, Huifang Hou, Xiayu Chen, Zhuoxian Zhao, Yinyue Deng

**Affiliations:** 1 School of Pharmaceutical Sciences (Shenzhen), Shenzhen Campus of Sun Yat-sen University, Sun Yat-sen University 626353 , Shenzhen, China; Indiana University Bloomington, Bloomington, Indiana, USA

**Keywords:** quorum sensing, *Burkholderia cepacia *complex, AHL, BDSF, virulence

## Abstract

Quorum sensing (QS) is a cell-cell communication mechanism widely employed by bacteria to control group behaviors in a cell density-dependent manner. QS plays a critical role in the regulation of physiological processes in the *Burkholderia cepacia* complex (Bcc), which consists of at least 28 closely related species. To date, several different QS systems have been identified in the Bcc, including the well-characterized *N*-acyl-L-homoserine lactone-type QS systems and diffusible signaling factor-type QS systems. Here, we review the research progress on QS in the Bcc, including biosynthesis, biological functions, and regulatory mechanisms. We compare the biosynthetic pathways and regulatory mechanisms of these QS signals, which reveal their specificity and universality. We also review recent antibacterial research, which focuses on targeting these QS signaling systems, and the application prospects of this strategy.

## INTRODUCTION

The *Burkholderia cepacia* complex (Bcc) is a group of closely related Gram-negative bacteria comprising at least 28 species (1–6). These species are characterized by extraordinary genetic diversity, adaptability, and robust environmental persistence, enabling ubiquitous colonization of diverse habitats—from soil, aquatic systems, and plant rhizospheres to hospital environments (2, 3, 7). Clinically, some Bcc members are formidable opportunistic pathogens, notorious for causing severe respiratory infections and sepsis in immunocompromised individuals (7, 8). Compounding this threat are their intrinsic multidrug resistance and ability to persist in chronic infections, which complicate therapeutic interventions and underscore the urgent need for novel antimicrobial strategies (9, 10).

Central to the pathogenic success of the Bcc is its regulatory network, especially the quorum sensing (QS) system. QS describes cell-cell communications that use small signals, such as *N*-acyl homoserine lactones (AHLs) and diffusible signal factor (DSF) signals, to synchronize gene expression in response to cell density (11–15). This process plays a pivotal role in coordinating bacterial behaviors critical for pathogenicity, such as biofilm formation, secretion of extracellular enzymes, and evasion of host immune defenses (16, 17). Furthermore, Bcc not only employs multiple QS systems that interact in a cascading manner but also integrates these systems with nucleotide second messengers, such as cyclic di-guanosine monophosphate (c-di-GMP) signaling, adding layers of complexity to its functional landscape (18–20). Intriguingly, certain Bcc strains possess atypical QS components or “orphan” regulators, suggesting evolutionary adaptations tailored to specific ecological niches (21, 22).

Given the crucial role of QS in bacterial virulence, there has been considerable interest in developing novel therapeutic strategies that specifically target and block QS systems. Such anti-virulence approaches represent a paradigm shift from traditional bactericidal strategies, potentially reducing the selective pressure for antibiotic resistance (23–27). Understanding the intricacies of QS signaling in the Bcc is essential for the development of novel therapeutic strategies and infection control measures. The aim of this review is to provide a comprehensive overview of current knowledge on the ecological distribution, infection outcomes, industrial applications, and taxonomic classification of Bcc; the synthesis and function of QS signals; QS-related sensing mechanisms and signal transduction pathways; and their regulatory mechanisms on virulence factor production and biofilm development.

## CLASSIFICATION AND OVERVIEW OF Bcc

In the mid-20th century, Professor Burkholder from Cornell University isolated a strictly aerobic, non-spore-forming, Gram-negative, rod-shaped microorganism from diseased onion bulbs (1). Initially named *Pseudomonas cepacia*, the bacterium was later reclassified and renamed *Burkholderia cepacia* and belongs to the genus *Burkholderia* (28, 29). Subsequent polyphasic taxonomic studies revealed that many strains phenotypically identified as *B. cepacia* actually comprised closely related genotypic subgroups. These subgroups form the Bcc (2).

To date, 28 Bcc species have been classified and named, including *B. cepacia, B. multivorans, B. cenocepacia, B. stabilis, B. vietnamiensis, B. dolosa, B. ambifaria, B. anthina, B. pyrrocinia, B. ubonensis, B. arboris, B. contaminans, B. diffusa, B. lata, B. latens, B. metallica, B. pseudomultivorans, B. seminalis, B. stagnalis, B. territorii, B. paludis, B. puraquae, B. catarinensis, B. aenigmatica, B. orbicola, B. semiarida, B. sola,* and *B. alpina* (Table 1). Among these, *B. cepacia* is the representative species (2–6, 30–32).

**TABLE 1:** Bcc species classification and characteristics

Species name	Genomovar^*a*^	Clinical relevance	Key characteristics	Type strain	Reference
*Burkholderia cepacia*	Genomovar I	Low	Environmental bacteria, plant pathogen	ATCC 25416	(33)
*B. multivorans*	Genomovar II	High	Common in cystic fibrosis infections, hospital-acquired	LMG 13010	(34)
*B. cenocepacia*	Genomovar III	High	High virulence, multidrug-resistant, strong biofilm formation	LMG 16656	(34)
*B. stabilis*	Genomovar IV	Low	Strong environmental adaptability, sporadic infections	LMG 14294	(35)
*B. vietnamiensis*	Genomovar V	Medium	Soil bacteria, occasionally causes lung infections	LMG 10929	(36)
*B. dolosa*	Genomovar VI	High	Cystic fibrosis infections, multidrug-resistant	LMG 18943	(37)
*B. ambifaria*	Genomovar VII	Low	Environmental bacteria, potential for biological control	LMG 19182	(38)
*B. anthina*	Genomovar VIII	Low	Environmental bacteria, plant interaction	LMG 20980	(39)
*B. pyrrocinia*	Genomovar IX	Low	Environmental bacteria, antibiotic production	LMG 14191	(40)
*B. ubonensis*	/	Low	Environmental bacteria, commonly found in soil	LMG 20358	(41)
*B. latens*	/	Low	Environmental bacteria, isolated from plant roots	LMG 24064	(42)
*B. diffusa*	/	Low	Environmental bacteria, isolated from soil	LMG 24065	(42)
*B. arboris*	/	Low	Environmental bacteria, isolated from plants	LMG 24066	(42)
*B. seminalis*	/	Medium	Isolated from clinical samples, sporadic infections	LMG 24067	(42)
*B. metallica*	/	Low	Environmental bacteria, isolated from metal-contaminated soil	LMG 24068	(42)
*B. contaminans*	/	Medium	Isolated from clinical samples, multidrug-resistant	LMG 23361	(43)
*B. lata*	/	Low	Environmental bacteria, isolated from soil	LMG 22485	(43)
*B. pseudomultivorans*	/	Medium	Isolated from clinical samples, similar to B. multivorans	LMG 26883	(44)
*B. territorii*	/	Low	Environmental bacteria, isolated from soil	LMG 28158	(45)
*B. puraquae*	/	Low	Environmental bacteria, isolated from water bodies	LMG 29660	(46)
*B. paludis*	/	Low	Environmental bacteria, isolated from wetlands	DSM 100703	(31)
*B. stagnalis*	/	Low	Environmental bacteria, isolated from water bodies	LMG 28156	(45)
*B. catarinensis*	/	Low	Environmental bacteria, isolated from soil	DSM 103188	(30)
*B. aenigmatica*	/	Medium	Isolated from clinical samples, such as respiratory tract or wound infections	LMG 13014	(47)
*B. orbicola*	/	Medium	Opportunistic pathogens, isolated from human infections	LMG 30279	(4)
*B. semiarida*	/	Low	Isolated from onion bulbs	IBSBF 3371	(5)
*B. sola*	/	Low	Isolated from onion bulbs	IBSBF 3372	(5)
*B. alpina*	/	Low	Isolated from an alpine altitude	LMG 28138	(6)

^*a*^ /, data not available or not applicable.

Bcc is ubiquitously distributed in natural environments, with its robust metabolic versatility enabling adaptation to diverse ecological niches. Its ecological range spans soil matrices, aquatic systems, plant rhizospheres, industrial facilities, and nosocomial settings, encompassing zoonotic pathogens, opportunistic human pathogens, phytopathogens, and environmental saprophytes (32, 48). Soil-derived Bcc strains, such as *B. cepacia, B. ambifaria, B. pyrrocinia,* and *B. cenocepacia,* can utilize complex and diverse carbon sources and are capable of degrading different compounds and organic pollutants (48). In purified water systems, *B. cepacia* exhibits persistent colonization through biofilm formation, a key survival strategy contributing to its recalcitrance to eradication in aqueous environments (49). Bcc isolates identified from plant inter-roots, such as *B. vietnamiensis, B. ambifaria,* and *B. pyrrocinia,* can be used as biocontrol agents for plant growth promotion and bioremediation applications after undergoing stringent biosafety evaluations or genetic modification (50). A proportion of Bcc isolates are also distributed in industrial environments, such as *B. cepacia*, which contaminates production equipment for cosmetics, pharmaceuticals, etc. (51, 52). The persistence of Bcc pathogens in hospitals is of particular clinical concern. Outbreaks involving *B. cepacia*, *B. multivorans,* and *B. cenocepacia* in critical care units, hemodialysis centers, and endoscopic suites have been extensively documented. These opportunistic pathogens pose severe threats to immunocompromised populations, particularly those causing life-threatening respiratory infections in people with cystic fibrosis (CF) or chronic granulomatous disease (CGD) (7, 53, 54).

### Bcc human pathogens

Over the past 2 decades, Bcc has emerged as a significant opportunistic pathogen in individuals with certain underlying conditions, particularly those with CF, CGD, and primary immunodeficiency. Bcc infection in people with CF frequently culminates in rapid pulmonary deterioration accompanied by septicemia, a clinical trajectory formally designated as “Cepacia syndrome” (55, 56). Many Bcc strains have been isolated and identified in the sputum of people with CF, including *B. cepacia, B. multivorans, B. cenocepacia, B. stabilis, B. vietnamiensis, B. dolosa, B. ambifaria, B. anthropi,* and *B. pyrrocinia*. In vulnerable populations, especially in individuals with potential diseases such as CF, these species can cause severe, persistent lung infections with potentially lethal outcomes due to their heightened virulence. These pathogens are resistant to known antibiotics and have been shown to spread among people with CF, posing a significant burden on them (32, 57). Beyond their association with CF, Bcc pathogens pose significant risks to individuals with CGD, where they can trigger life-threatening systemic infections. Notably, Bcc-induced sepsis and pneumonia are recognized as major determinants of mortality in the CGD population (58, 59).

### Bcc plant pathogens

*B. cepacia* is also a plant pathogen that can cause onion acid skin disease. It carries genes encoding a pectinase—endopolygalacturonase, an enzyme that hydrolyzes α-1,4-glycosidic linkages within the polygalacturonic acid chains of pectin in the middle lamella and primary cell walls (60). The pathogen exists in soil or irrigation water and invades bulb tissues through wounds in crops or is washed from the leaves onto the bulb scales. Under postharvest conditions characterized by warm temperatures (25°C–30°C) and acidic environments (pH 5.0–6.0), *B. cepacia* synthesizes endopolygalacturonase, resulting in water-soaked lesions in onion tissues, damaging the scale tissues and causing fluid accumulation (referred to as Plant Tissue Water-Soaking, PTW) (60). Both *B. cenocepacia* and *B. vietnamiensis* can also cause PTW symptoms, as they both contain a plasmid encoding a type IV secretion system, which secretes water-soluble plant cell toxic factors (61). By conducting in-depth research on the biological characteristics of Bcc plant pathogenic bacteria, environmentally friendly biological disease prevention products can be developed, while providing new ideas for sustainable management of agricultural ecosystems.

### Bcc plant growth-promoting rhizobacteria

It has been documented that various Bcc isolates, including *B. cepacia, B. ambifaria, B. pyrrocinia,* and *B. cenocepacia,* can colonize plant roots in substantial numbers and possess multiple growth-promoting functions (62). *B. vietnamiensis* can convert atmospheric nitrogen into ammonia, promoting nitrogen cycling and increasing nitrogen input in agricultural ecosystems (36). *B. contaminans* and *B. lata* have also been demonstrated to enhance plant nitrogen absorption and utilization through biological nitrogen fixation (63). Furthermore, *B. cepacia* has been identified as capable of transforming insoluble phosphorus in soil into soluble forms through the secretion of organic acids or acid phosphatases, significantly improving crop phosphorus uptake from the soil and promoting plant growth. Experimental results demonstrated that plants inoculated with this strain exhibited a 44% higher phosphorus content compared to the uninoculated control group (64). *B. cepacia* and *B. ambifaria* can promote plant growth by producing aminocyclopropane-1-carboxylate (ACC) deaminase, which breaks down ACC, a precursor of ethylene, into ammonia and α-ketobutyrate (65, 66). *B. ambifaria* T16 can also produce the plant growth hormone indole-3-acetic acid to stimulate plant root growth (66). The application of selected beneficial Bcc strains in the development of green crop production systems, following rigorous biosafety assessment, can effectively reduce the use of chemical fertilizers and pesticides, promoting agriculture and eco-friendly development.

### Bcc biodegradation bacteria

The Bcc represents a phylogenetically diverse consortium of bioremediation agents with demonstrated efficacy in attenuating recalcitrant environmental pollutants (67, 68). Of particular environmental toxicological relevance, trichloroethylene (TCE), a pervasive groundwater contaminant in US aquifers, is efficiently degraded by specific *Burkholderia* species via cometabolic pathways (69). *B. vietnamiensis* can metabolize TCE in the presence of aromatic inducers such as toluene or phenol. Field inoculation experiments have shown that *B. vietnamiensis* ENV435 can reduce the content of chlorinated solvents in sandy layers by 70%, effectively helping mitigate soil pollution (70). Dichloroquinolinic acid is a selective herbicide used to control barnyard grass in fields, and its excessive use can easily cause crop phytotoxicity. In 2003, Lü et al. isolated and screened a high-efficiency dichloroquinolinic acid-degrading bacterium, *B. cepacia* WZ1, from soil near a pesticide factory, with a degradation activity as high as 90% (71). Additionally, *B. vietnamiensis* G4 can degrade multiple aromatic hydrocarbons, including benzene, cresol, o-cresol, phenol, toluene, and TCE (72, 73). Collectively, these studies demonstrate that Bcc has significant application potential in degrading recalcitrant aromatic hydrocarbons and in ecological environmental restoration.

## THE AHL-TYPE QS IN Bcc

QS was first discovered in the marine bacterium *Vibrio fischeri*, which uses QS signals to regulate bioluminescence in Hawaiian squid, providing them with camouflage (74, 75). It was revealed that two key components orchestrate QS: LuxI and LuxR (76). LuxI is an autoinducer synthase that produces *N*-acyl-L-homoserine lactone (AHL) signaling molecules. LuxR, a cytoplasmic receptor and transcription factor, binds accumulated AHLs at high cell density. The LuxR-AHL complex then activates transcription of luciferase genes by binding target promoters. This LuxIR paradigm is widespread in Gram-negative bacteria (77). The Bcc possesses AHL-type QS systems, which not only are crucial for environmental adaptation and opportunistic infections but also serve as potential targets for anti-infection therapies.

AHL-type QS representative systems in Bcc include CepIR, CciIR, and BviIR (12, 78, 79). The CepIR system regulates functions such as protease production and biofilm formation (15, 80–84). In this system, CepI synthesizes *N*-octanoyl-homoserine lactone (C8-HSL, OHL) and a small amount of *N*-caproyl-homoserine lactone (C6-HSL, HHL), while CepR acts as the corresponding receptor protein (78, 85). The CciIR system is found exclusively in *B. cenocepacia*, interacting with the CepIR system to regulate motility and protease production (virulence factor) (79). The BviIR system is unique to *B. vietnamiensis* and can produce various AHL signaling molecules, such as C8-HSL, C6-HSL, *N*-decanoyl-homoserine lactone (C10-HSL, DHL), *N*-dodecyl-homoserine lactone (C12-HSL), and *N*-3-oxodecanoyl-homoserine lactone (3OC10-HSL) (86). Notably, *B. multivorans* typically produces little to no detectable AHLs under standard conditions, despite possessing the AHL synthase BmuI and transcriptional activator BmuR (85, 87). However, AHL-overproducing mutants of *B. multivorans* have been isolated, which were found to produce multiple AHLs, including C6-HSL, C8-HSL, and additional unidentified AHL species. The mutations leading to this overproduction were located outside the *bmuIR* locus, suggesting the existence of unknown upstream regulators that repress the QS system in the wild-type strains 17616-FY2 and 17616-A3 (88, 89). Additionally, other AHL QS-related factors in Bcc have been identified, such as CepR2 (21), BCAM1817 (90), ShvR (91), RsaM (92), and AtsR (93).

### The biosynthesis of AHL signals

**Fig 1:**
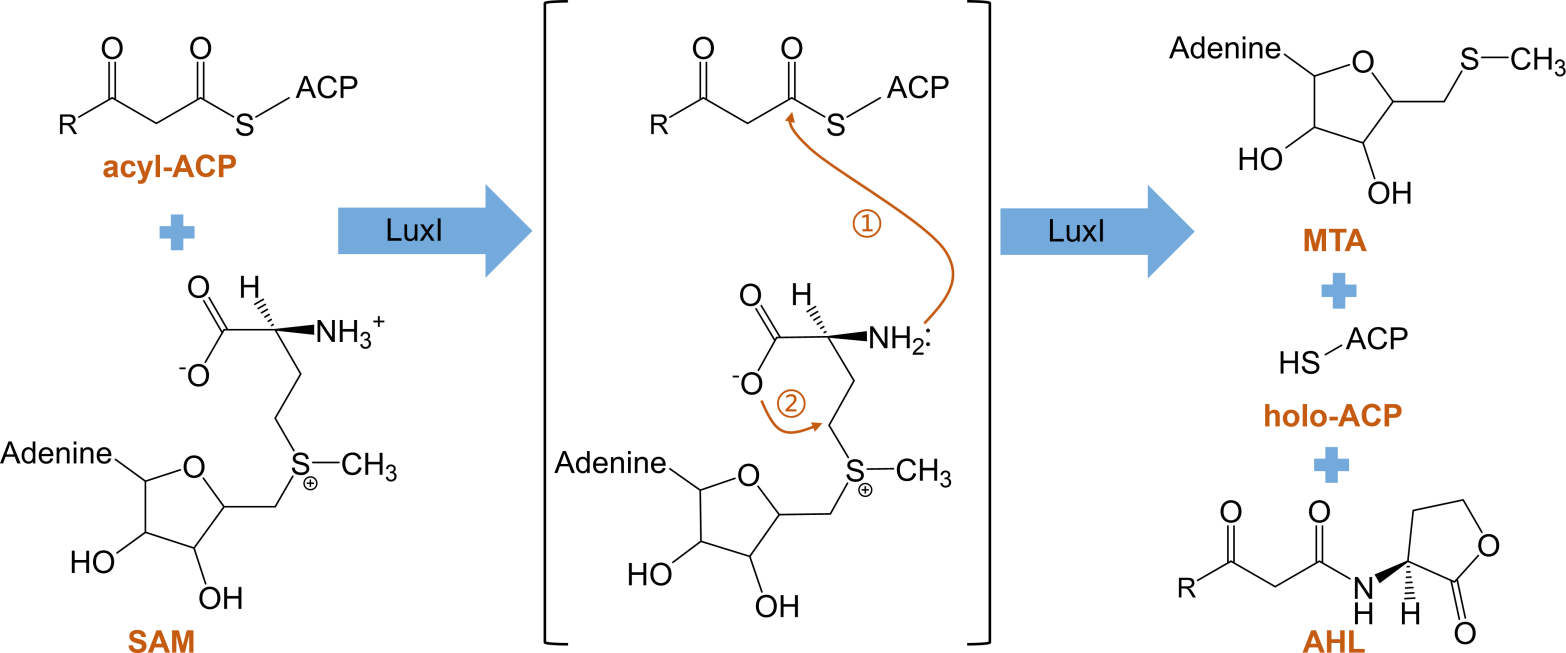
Schematic diagram of AHL synthesis. AHL is synthesized from acyl-ACP and SAM through the action of LuxI. ① The AHL synthase LuxI transfers the acyl side chain from acyl-ACP to the homocysteine moiety of SAM. ② The acylated HSL molecule moiety then undergoes lactonization, generating holo-ACP, 5'-methylthioadenosine (MTA), and AHL.

Many Gram-negative bacteria possess LuxI-type autoinducer synthases capable of producing AHL signals, which are the most studied autoinducer molecules. The core of the AHL-type signal is an L-homoserine lactone ring, with an acyl chain length of 4-18 carbon atoms (94, 95). The LuxI enzyme catalyzes the transfer of an acyl group from the acyl carrier protein (acyl-ACP) to the homocysteine moiety of S-adenosylmethionine (SAM), producing a specific acylated homoserine lactone (HSL). This HSL then undergoes lactonization to form acyl-homoserine lactone (acyl-HSL), commonly referred to as AHL (Fig. 1). AHL signaling molecules are classified on the basis of acyl chain length into short-chain AHL signals (4–8 carbons) and long-chain AHL signals (10–18 carbons). Short-chain AHL signals rely primarily on passive diffusion for transport, whereas long-chain AHL signals are actively transported (96).

### The signaling pathways of AHL-type QS signals in Bcc

#### The signaling pathway of the CepIR system

**Fig 2:**
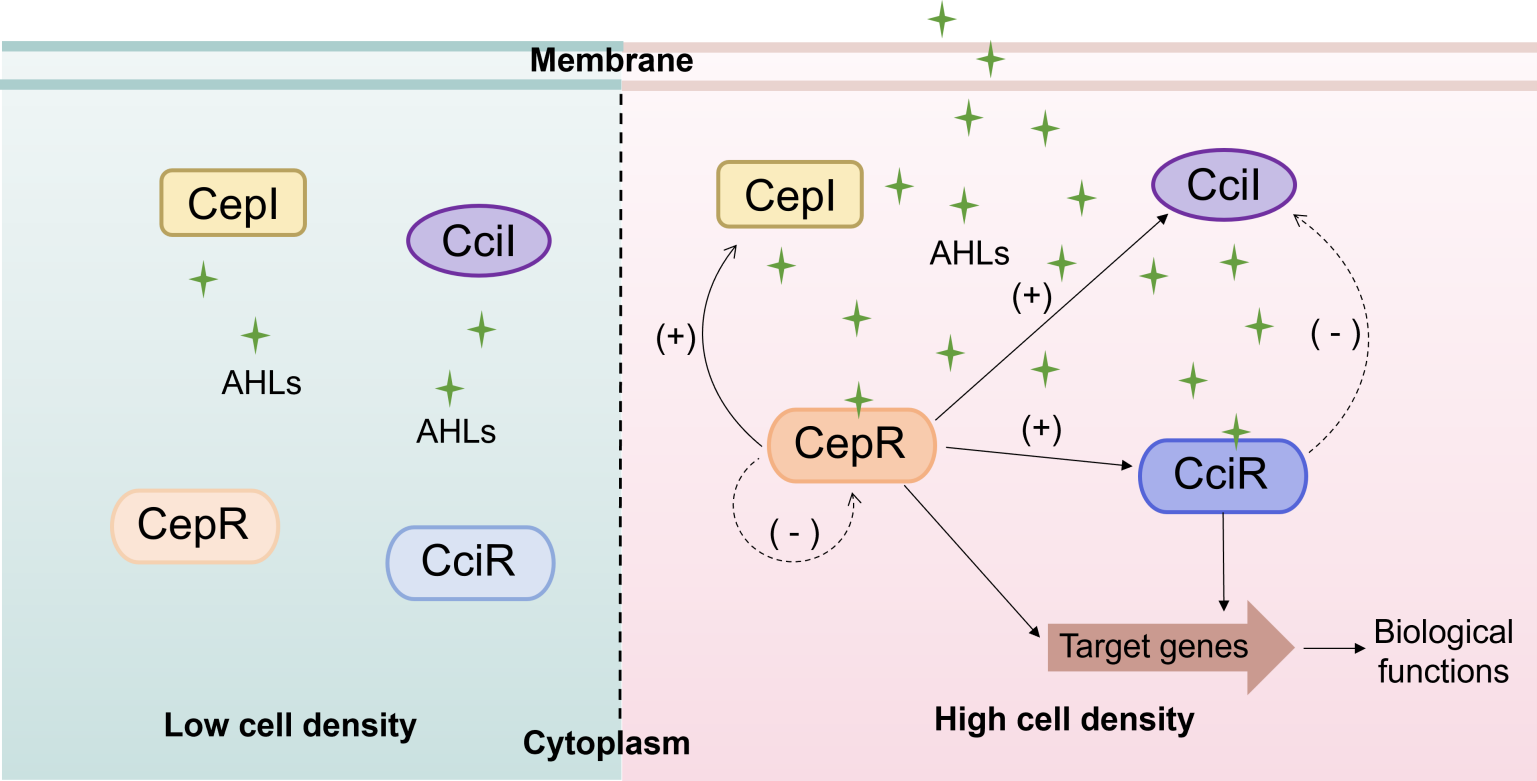
Schematic diagram of AHL-mediated QS regulatory networks composed of the CepIR and CciIR systems in the Bcc. AHLs are produced by the AHL synthases CepI and CciI. At high cell density, accumulated AHLs bind to the transcriptional regulators CepR and CciR, forming CepR-AHL and CciR-AHL complexes, respectively. These complexes activate the expression of target genes, thereby regulating biological functions. The CepR-AHL complex positively regulates *cepI* transcription to amplify AHL production, while negatively regulating *cepR* expression. The CciR-AHL complex exerts negative feedback on *cciI* expression. In addition, the CepIR system positively regulates both *cciI* and *cciR* genes.

In 1999, a homologous system of LuxIR was identified in *B. cenocepacia* K56-2 and designated CepIR. Subsequent studies confirmed that the CepIR system is conserved across Bcc (78). The CepIR system comprises two functional proteins: the AHL synthase CepI and an AHL-dependent transcriptional regulator CepR that binds target promoters to activate gene expression upon signal binding (12, 85, 97). CepI primarily synthesizes C8-HSL and a relatively small amount of C6-HSL (78, 85). Like LuxR, CepR contains two distinct domains: an N-terminal AHL-binding domain and a C-terminal DNA-binding domain. At low cell density, basal levels of AHLs are produced via CepI, diffuse out of the cell, and accumulate in the environment. At high cell density, once the AHL concentration reaches a threshold, it binds to CepR, inducing a conformational change in the protein. This complex then binds to the promoters of target genes, regulating their expression (Fig. 2).

The CepIR system regulates various biological functions in Bcc, including positive regulation of protease production, biofilm formation, and swarming motility, and negative regulation of siderophore production (15, 80–84). Additionally, the CepIR system positively regulates polygalacturonase production and negatively regulates the σ^s^ factor-encoding gene *rpoS* (98). The CepIR system also exhibits autoregulation: when AHL signals bind to CepR, the resulting complex binds to the *cep* box near the −35 promoter region of *cepI*, thereby positively regulating the transcription of *cepI* to amplify AHL signal production and accelerate population-wide response; while the complex also negatively regulates the transcription of *cepR,* which may serve to slow down the QS response once population density declines (82, 97).

#### The signaling pathway of the CciIR system

In addition to the CepIR system, a distinct CciIR system has been identified in *B. cenocepacia*. The CciIR system was discovered during an investigation of the *B. cepacia* epidemic strain marker (BCESM) gene cluster (79). BCESM is part of a 31.7 kb genomic island known as the cenocepacia island (cci), which harbors the *cciI* and *cciR* genes. This system includes an AHL synthase gene (*cciI*) and a predicted response regulator gene (*cciR*) (99). CciI facilitates the synthesis of C6-HSL as the primary product, with a minor amount of C8-HSL, which is different from that of CepI.

The CciIR system has a negative regulatory effect on *cciI* expression but has no discernible effect on *cciR* expression. The CepIR system, situated upstream of the CciIR system, regulates the expression of both the *cciI* and *cciR* genes. Moreover, the CciIR system has been shown to positively regulate motility and protease production through AHL production by CciI (79). Phylogenetic evidence indicates that horizontal gene transfer from a distal taxon introduced the CciIR system, which was later co-opted into the pre-existing CepIR regulatory network during evolution. The CepIR and CciIR QS systems transcriptionally regulate multiple genes in *B. cenocepacia,* including potential virulence genes associated with motility, biofilm formation/adhesion, secretion systems, and so on. The CepIR system primarily exerts positive regulation on co-regulated QS targets, such as motility genes, the nematocidal protein AidA, the *shvR*/*afcA* genomic region, type VI secretion systems, heat- and cold-shock proteins, Flp-type pilus components, the fimbrial protein FimA, and lectins. In contrast, the CciIR system generally mediates negative regulation of these targets. Notably, a minority of co-regulated genes (e.g., ferric ornibactin transport systems) exhibit positive regulation by CciIR (79, 100).

#### The signaling pathway of the BviIR system

Examination of the BviIR system in *B. vietnamiensis* revealed that, in addition to the *cepI* gene, this bacterium also possesses another autoinducer synthase gene, *bviI*, along with its corresponding regulatory gene, *bviR*. This system is unique to *B. vietnamiensis* within Bcc (12). *B. vietnamiensis* produces a diverse array of AHL signaling molecules, including C8-HSL, C6-HSL, C10-HSL, C12-HSL, and 3OC10-HSL (86). The BviI synthase is responsible for the production of long-chain AHLs (C10-HSL and C12-HSL), while the CepI synthase produces C6-HSL and C8-HSL (101). The expression of *bviI* requires the joint regulation of both BviR and CepR, whereas BviR does not influence the CepIR system (22). Furthermore, the BviIR system does not participate in siderophore production (86). Owing to compensation by other systems, phenotypic differences in BviIR system mutants are difficult to detect, resulting in limited research on related phenotypes.

#### Other AHL QS-related factors in Bcc

The AHL synthase gene and its receptor gene are typically located in close proximity within the genome. Genome-wide analyses of Gram-negative bacteria have revealed that LuxR homologs are more abundant than LuxI homologs. Although many LuxR proteins are not associated with AHL synthases, they still require AHL molecules for their functional activity (102, 103). In *B. cenocepacia*, CepR2 was identified as an “orphan” LuxR homolog unassociated with an adjacent *N*-acyl-homoserine lactone synthase gene (21). In *B. cenocepacia* K56-2, CepR2 undergoes ligand-independent negative autoregulation: CepR2 monomers bind a palindromic operator upstream of *cepR2* to repress transcription. This occurs concurrently with CciR-mediated repression, collectively constraining orphan receptor production under non-inducing conditions. CepR2 does not affect the expression of *cepI* and *cepR* or that of *cciI* and *cciIR*. Furthermore, CepR2 negatively regulates the expression of several QS-controlled genes, including genes encoding zinc metalloproteases (21). Additionally, Ryan et al. revealed that several *cepR2*-linked promoters are repressed by CepR2, which is antagonized by AHL signaling molecules. When expressed in *E. coli*, CepR2 represses the promoters of *BCAM0191* and *BCAM0192* (104). Additionally, CepR2 can activate the transcription of target promoters in the absence of AHL signaling molecules. In a heterologous system, the ability of CepR2 to activate the *luxI* promoter remained unaffected even after the addition of ten different AHLs, including OHL (6). Notably, a homologous *cepR2* gene exists in *B. ambifaria*, but its genomic organization differs significantly: while *B. ambifaria* encodes a LuxI synthase (Bamb_6053, annotated as CepI2), this synthase is located distally from *cepR2* on a separate genomic region, indicating species-specific divergence in QS module architecture (104, 105).

Other regulatory proteins associated with the AHL-mediated QS have been identified in *B. cenocepacia*. BCAM1817, located downstream of the *cepI* gene, encodes a hypothetical conserved protein that can increase AHL production to positively regulate the expression of the CepIR and CciIR systems and activate the expression of ShvR, the LysR-type transcriptional regulator (90, 91). The *shvR* gene is highly conserved in Bcc and chromosomally located adjacent to the *afc* antifungal cluster (106). It drives lung inflammation in rat chronic infection models and regulates key virulence genes through dual mechanisms: *zmpA* and *zmpB* encode zinc metalloproteases that degrade host tissues and exacerbate chronic respiratory infections, while *afcA* and *afcC* form the core of an antifungal biosynthesis operon that inhibits phytopathogens such as *Rhizoctonia solani* (91, 106). By coordinating these functions, ShvR integrates QS signaling with type II secretion and antifungal defense pathways (91). RsaM, a structurally unique single-domain protein, is involved in regulating the expression of the *cepIR* gene. However, no known DNA-binding motifs have been identified in studies, and how RsaM regulates the QS system remains to be further investigated (92). Another regulatory protein, AtsR, negatively regulates the expression of *cepI* and *cciI* and affects QS-regulated biological phenotypes, including biofilm formation, protease production, swarming motility, and the type VI secretion system (93). The interactions between AtsR and other regulatory proteins affecting the QS system in Bcc remain to be elucidated.

## THE DSF-TYPE QS IN Bcc

In addition to AHL signals, the DSF signals represent another ubiquitous class of QS signaling molecules in Gram-negative bacteria (107). Experimental evidence has demonstrated that DSF-mediated QS orchestrates multiple virulence-associated phenotypes, including biofilm formation, flagellum-driven motility, and exoenzyme biosynthesis (14, 108–110).

Notably, in 2008, Boon et al. identified a distinct DSF-family signal termed BDSF in *B. cenocepacia* J2315 (23). Using high-performance liquid chromatography and mass spectrometry, Deng et al*.* detected BDSF signals in all nine tested Bcc species (*B. cenocepacia*, *B. lata*, *B. ambifaria*, *B. stabilis*, *B. dolosa*, *B. anthina*, *B. pyrrocinia*, *B. vietnamiensis*, and *B. multivorans*). Among these, *B. anthina*, *B. stabilis*, and *B. pyrrocinia* produced not only BDSF but also another DSF-family compound, *cis*-11-methyl-dodec-2,5-dienoic acid. In *B. multivorans*, the presence of the DSF synthase BmuI5121 was associated with the production of both these molecules, along with DSF (17). As a conserved signaling molecule within Bcc, BDSF structurally diverges from the canonical DSF, lacking a methyl group at the C-11 position (23). Importantly, the BDSF signaling cascade and its regulatory network exhibit marked specificity and mechanistic divergence compared with established DSF systems.

### Synthesis of the BDSF signal

**Fig 3:**
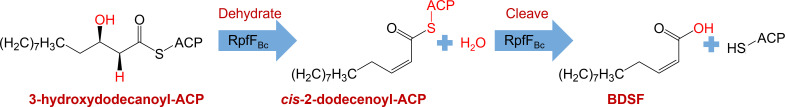
RpfF_Bc_-catalyzed biosynthesis pathway of BDSF. The BDSF signaling molecule is synthesized by RpfF_Bc_, a bifunctional enoyl hydratase possessing both dehydratase and thioesterase activities. The synthesis proceeds via two enzymatic steps: RpfF_Bc_ first catalyzes the conversion of 3-hydroxydodecanoyl-ACP into *cis*-2-dodecenoyl-ACP. Subsequently, RpfF_Bc_ releases the free fatty acid *cis*-2-dodecenoic acid (BDSF) by cleaving the thioester bond in *cis*-2-dodecenoyl-ACP.

In *B. cenocepacia*, BDSF is synthesized by RpfF_Bc_ (a homolog of RpfF, originally identified in *Xanthomonas campestris* as an enoyl-CoA hydratase/isomerase essential for DSF signal production) (111, 112). RpfF_Bc_ is encoded by *BCAM0581* in *B. cenocepacia* J2315. This protein is a bifunctional enoyl hydrolase that has both dehydrase and sulfatase activities. The dehydrase activity converts the ACP of 3-hydroxydecanoic acid to a *cis*-2-dodecyl-ACP, which is then cleaved by the sulfatase activity to release free *cis*-2-dodecenoic acid (Fig. 3) (111). The RpfF_Bc_ homolog is not only widely present in the Bcc but also found in other genera, including *Achromobacter*, *Yersinia*, *Serratia*, *Enterobacter*, *Pantoea*, *Cronobacter*, *Rahnella*, *Erwinia,* and *Yokenella* (18). Like AHLs, BDSF accumulates in a cell density-dependent manner, with the highest levels observed during the stationary phase.

### The signaling pathways of the BDSF QS systems in Bcc

#### The signaling pathway of the RpfF_Bc_-BCAM0227 system

McCarthy et al. identified a novel BDSF receptor protein, BCAM0227, in *B. cenocepacia* J2315 (113). BCAM0227 shares 35.6% homology with RpfC from *Xanthomonas campestris* pv. campestris (Xcc). In Xcc, DSF is synthesized by RpfF and subsequently sensed and transduced by the two-component signaling system RpfC-RpfG. RpfC, functioning as the DSF receptor, comprises four distinct domains: a histidine kinase phosphotransfer domain (HisKA), a histidine kinase ATP-binding domain (HATPase_c), a two-component receiver domain (REC), and a C-terminal histidine phosphotransfer domain (HPT) (112, 114). Notably, while BCAM0227 also has these domains, the HPT domain in BCAM0227 is not identified by the SMART database (Simple Modular Architecture Research Tool) but is annotated as a Pfam domain. Another notable difference lies in their predicted membrane topologies: RpfC is predicted to contain five transmembrane helices, whereas BCAM0227 possesses only two transmembrane helices localized in the bacterial inner membrane. Importantly, deletion of the *BCAM0227* gene affected the expression of a subset of BDSF-regulated genes. Both *BCAM0227* and *rpfF_Bc_* were found to influence biofilm formation, but significant differences in pathogenic phenotypes—such as motility and mucin adhesion—were observed between *BCAM0227* and *rpfF_Bc_* mutants (113).

#### The signaling pathway of the RpfF_Bc_-RpfR system

**Fig 4:**
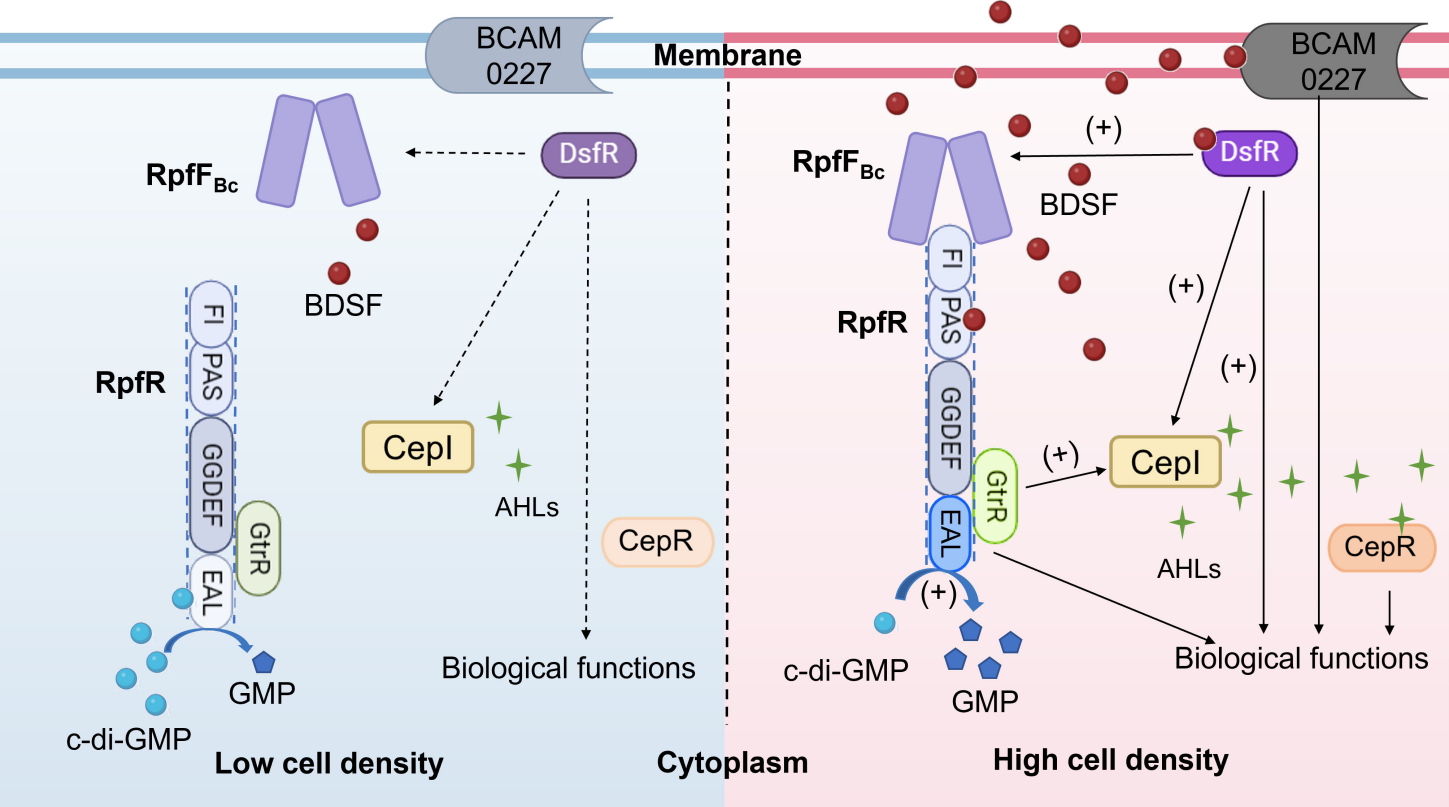
The signaling pathway of the BDSF-type QS systems. RpfF_Bc_ is responsible for synthesizing the BDSF signal, while BCAM0227, RpfR, and DsfR serve as the BDSF receptor proteins. BCAM0227 is a transmembrane protein. BDSF is sensed and transduced by BCAM0227 to regulate partial biological functions when at high cell density; RpfR contains three functional domains: an FI-PAS domain, a GGDEF domain, and an EAL domain. When bacterial density reaches a certain threshold, the PAS domain of RpfR binds to BDSF, enhancing PDE activity. Reduction of c-di-GMP levels activates the RpfR-GtrR complex to modulate gene expression. Furthermore, activated RpfR interacts with RpfF_Bc_ through its FI domain, thereby inhibiting BDSF synthesis; DsfR regulates the transcription of target genes by directly binding to gene promoters. When bacterial density reaches a certain level, BDSF binds to DsfR, enhancing the expression of AHL and BDSF signal synthase-encoding genes *cepI* and *rpfF_Bc_*, thereby increasing the synthesis of BDSF and AHL signals. The increased AHL levels activate their receptor CepR to control the transcription of target genes.

Deng et al. reported the BDSF receptor protein RpfR (18). The *rpfR* gene (*BCAM0580* in the genome of *B. cenocepacia* J2315) is located near *rpfF_Bc_* in the genome. RpfR contains FI-PAS, GGDEF, and EAL domains (Fig. 4) (115). The PAS domain has been reported to bind small molecules with a range of chemical characteristics, including heme, flavin, divalent metal cations, amino acids, and coumaric acid (116). The GGDEF and EAL domains are two highly conserved structures that encode c-di-GMP diguanylate cyclases (DGCs) and phosphodiesterases (PDEs), respectively, and their activities are related to the synthesis and degradation of c-di-GMP (117, 118). Studies have shown that in *B. cenocepacia* H111, deletion of the *rpfR* gene results in similar phenotypic changes to those of the *rpfF_Bc_* gene deletion mutant. Both mutants exhibit reduced swarming motility, decreased biofilm production, weakened proteolytic activity, and reduced extracellular polysaccharide production. When the BDSF signal is externally added to the *rpfF_Bc_* deletion mutant, the biological phenotype defective in the *rpfF_Bc_* mutant can be completely restored; however, the *rpfR* deletion mutant does not respond to BDSF signals. These findings indicate that RpfR is a downstream regulatory factor in the RpfFR system. Moreover, deletion of the *rpfF_Bc_* gene does not affect the transcription level of *rpfR*, suggesting that BDSF may influence the activity of RpfR through ligand-protein interactions. This hypothesis was confirmed by isothermal titration calorimetry and circular dichroism analysis, which revealed that BDSF binds to RpfR with high affinity *in vitro* and induces conformational changes, with the PAS domain being essential for BDSF binding (18).

RpfR is responsible for sensing and transmitting the BDSF signal. When the BDSF signal concentration reaches a threshold level, the molecules bind to RpfR and stimulate and activate the c-di-GMP PDE activity of RpfR, which in turn reduces the intracellular c-di-GMP levels. Changes in the intracellular c-di-GMP concentration lead to changes in the transcription levels of downstream target genes, thereby regulating various biological functions (Fig. 4) (18, 119, 120). C-di-GMP is a second messenger that has been extensively studied and regulates numerous bacterial biological functions in bacteria, including extracellular polysaccharide production, motility, and protease production (115, 117, 118, 121, 122). The concentration of intracellular c-di-GMP is regulated by DGCs and PDEs (19, 115). DGCs synthesize c-di-GMP from two GTP molecules, whereas PDEs hydrolyze c-di-GMP into linear pGpG or GMP. The GGDEF motif is essential for the enzymatic activity of DGCs, whereas PDE activity is associated with the EAL and HD-GYP domains (110, 118, 123–125). The phenotype of the *rpfR* gene deletion mutant in *B. cenocepacia* cannot be rescued by complementation with only the GGDEF (DGC) domain, but expression of the EAL (PDE) domain restores various pathogenicity-related phenotypes, such as protease production, biofilm formation, flagellar motility, and virulence, to wild-type levels (18).

Yang et al. demonstrated that RpfR not only has c-di-GMP PDE activity but can also serve as a c-di-GMP sensor. Their research identified a global regulator, GtrR (*BCAL1536* in the *B. cenocepacia* J2315 genome), which is a key downstream component that interacts with RpfR to regulate gene expression in *Burkholderia* (20). The RpfR-GtrR complex also regulates the transcription of *cepI*, promoting AHL synthesis and subsequently affecting the regulatory activity of CepR and associated biological functions. The deletion of the *cepIR* system does not affect the production of BDSF (126, 127). After the addition of exogenous c-di-GMP, the ability of the RpfR-GtrR complex to bind to the promoter DNA of target genes decreased, which was caused by c-di-GMP binding to RpfR. At low population densities, when the amount of BDSF is low and c-di-GMP exists at the basal level in the cells, c-di-GMP binds to the RpfR-GtrR complexes, preventing GtrR from activating transcription. At high population densities, when the concentration of BDSF exposed to the cells is relatively high, BDSF binds RpfR to stimulate its c-di-GMP PDE activity, thereby reducing the c-di-GMP level and increasing the regulatory activity of the RpfR-GtrR complex. Activated RpfR inhibits BDSF synthesis by interacting with RpfF_Bc_. This regulatory mechanism helps maintain the stability of the BDSF concentration. The PAS domain at the N-terminus of RpfR contains a highly conserved and uncharacterized region, FI. The FI domain of RpfR can directly bind to RpfF_Bc_, inhibiting BDSF synthesis (115). Furthermore, homologs of RpfR and GtrR are found in different Gram-negative bacteria and are conserved (homolog identity ≥95%) within the Bcc species, including *B. anthina*, *B. seminalis*, *B. cepacia*, *B. pyrrocinia*, *B. lata*, *B. contaminans*, and *B. stabilis*, indicating that the RpfF_Bc_-RpfR BDSF QS system might be widespread, particularly among Bcc pathogens (20).

Additionally, Cui et al. (128) discovered a new two-component system, RqpSR, where RqpS (*BCAL0535* in the *B. cenocepacia* J2315 genome) is a histidine kinase for signal transduction, and RqpR (*BCAL0534* in the *B. cenocepacia* J2315 genome) is a transcriptional regulator with a DNA-binding domain. This system not only controls the QS-regulated phenotypes of *B. cenocepacia* but also positively regulates the production of BDSF and AHL signals by modulating the transcription of *cepI* and *rpfF_Bc_* (128). The regulator of this system, RqpR, controls the QS system by directly binding to the promoters of the genes encoding BDSF and AHL signal synthases. These findings suggest that the RqpSR system regulates the physiological functions and pathogenicity of *B. cenocepacia* through complex hierarchical interactions with the QS system. Remarkably, orthologues of both RqpS and RqpR are highly conserved across *Burkholderia*, *Paraburkholderia*, and *Caballeronia* spp., where they consistently coexist with the widely distributed BDSF signal synthase. These orthologs exhibit substantial sequence similarity, particularly among *Burkholderia* species. Strikingly, RqpR orthologues in *B. cepacia* and *B. contaminans* share 99.1% identity with the reference RqpR protein (128).

The value of studying the mechanism of the QS system is reflected in the expression of its biological function. In *B. cenocepacia* J2315, the *rpfF_Bc_* mutant presented reduced toxicity and functional defects, including weakened swarming motility, decreased mucin adhesion, and reduced biofilm production. The mutant also shows diminished virulence in zebrafish infection models and *Galleria mellonella* larvae infection models (119, 120). The *bclACB* and *bapA* genes are closely associated with biofilm formation. In the *cepI rpfF_Bc_* double mutant, expression of the lectin BclB—encoded by the last gene of the *bclACB* locus—was found to be strongly dependent on BDSF. The expression of *bapA*, which is regulated by both AHL and BDSF signaling systems, was not restored to wild-type levels by exogenous addition of AHLs to the BDSF-deficient mutant (127). Additionally, in BDSF synthase-deficient mutants, the expression levels of virulence factors such as the metalloproteases ZmpA and ZmpB are reduced (119, 127, 129, 130).

#### The signaling pathway of the RpfF_Bc_-DsfR system

Li et al. identified the acyl-CoA ligase DsfR (*BCAM2136* in the *B. cenocepacia* J2315 genome) as one of the receptors of BDSF (131). DsfR regulates target gene transcription by directly binding to the gene promoter, and mutations in the leucine zipper structure of DsfR prevent its binding to the target gene promoter, indicating that the leucine zipper motif in DsfR is responsible for the binding of transcriptional regulators to target gene promoters. Additionally, BDSF binds to DsfR with high affinity and enhances the binding of DsfR to the promoter DNA regions of target genes. Two amino acid residues, Thr191 (T191) and Lys196 (K196), were found to be crucial for the interaction between DsfR and BDSF. These results indicate that DsfR is the specific receptor of BDSF in *B. cenocepacia*. Furthermore, the homologous protein RS02960 from *B. lata* 383 also bound to the *bclACB* promoter probe, and the perception of BDSF enhanced the binding activity of RS02960 (131).

In addition to directly binding to the promoters of downstream target genes to regulate their expression, DsfR can influence phenotypes in *B. cenocepacia* by modulating the production of AHL and BDSF signals (131). Specifically, the deletion of *dsfR* reduces the expression of *cepI* and *rpfF_B_*_c_, the genes encoding the AHL and BDSF QS signal synthases. The experimental results demonstrated significantly decreased production of C6-AHL, C8-AHL, and BDSF in the *dsfR* mutant, whereas the content of c-di-GMP increased (Fig. 4). This finding indicates that DsfR has a dual function in the QS regulatory network: on the one hand, DsfR acts as a BDSF receptor and can directly regulate the expression of downstream target genes and affect related physiological phenotypes; on the other hand, DsfR can simultaneously regulate the production processes of two QS signals (BDSF and AHL), thereby forming a complex regulatory network. This multilevel regulatory mechanism reveals the important role of DsfR in QS regulation and provides a new perspective for further research on the molecular mechanisms of the QS system.

Furthermore, as an acyl-CoA ligase, DsfR plays a crucial role in intermediary metabolism by converting fatty acids to fatty acyl-CoA, which is essential for basic cellular processes such as protein transport, enzyme activation, and phospholipid biosynthesis. These results suggest that DsfR has multiple important functions in *B. cenocepacia* (131).

### BDSF acts as an interspecies and inter-kingdom communication signal

In addition to intraspecies communication, BDSF can mediate interspecies interactions in polymicrobial environments where Bcc pathogens commonly reside, such as CF lungs and chronic wounds. Studies have shown that exogenous addition of BDSF can inhibit the transcription of QS-related genes and the production of QS signals in *P. aeruginosa*, including 3-oxo-C12-HSL, *Pseudomonas* quinolone signal (PQS, 2-heptyl-3-hydroxy-4-quinolone), and C4-HSL, thereby disrupting biofilm formation and virulence factor expression in *P. aeruginosa* (132). Other studies have shown that BDSF and some of its derivatives inhibit the type III secretion system (T3SS) of *P. aeruginosa*, indicating that BDSF may interfere with the QS system and T3SS of *P. aeruginosa* through two independent signaling pathways (132). However, the specific mechanisms by which BDSF interferes with the QS system and T3SS of *P. aeruginosa* still require further investigation. Moreover, BDSF inhibits the morphological transition of *Candida albicans* at physiologically relevant concentrations, thus blocking fungal biofilm formation (23, 133). Real-time reverse transcription quantitative PCR analysis has shown that BDSF can affect the expression of genes related to adhesion in *C. albicans*, such as *ALS1*, *EAP1*, and *YWP1* (134). Additionally, in a mouse infection model, BDSF can inhibit the expression of virulence factors in *C. albicans*, thereby exerting a protective effect (135). These findings further confirm the clinical application potential of QS signaling and its pathways.

## OTHER QS-RELATED SIGNALS IN Bcc

### Heptyl-4(1H)-quinolone (HHQ) and 4-Hydroxy-3-methyl-2-alkylquinolines (HMAQs)

**Fig 5:**
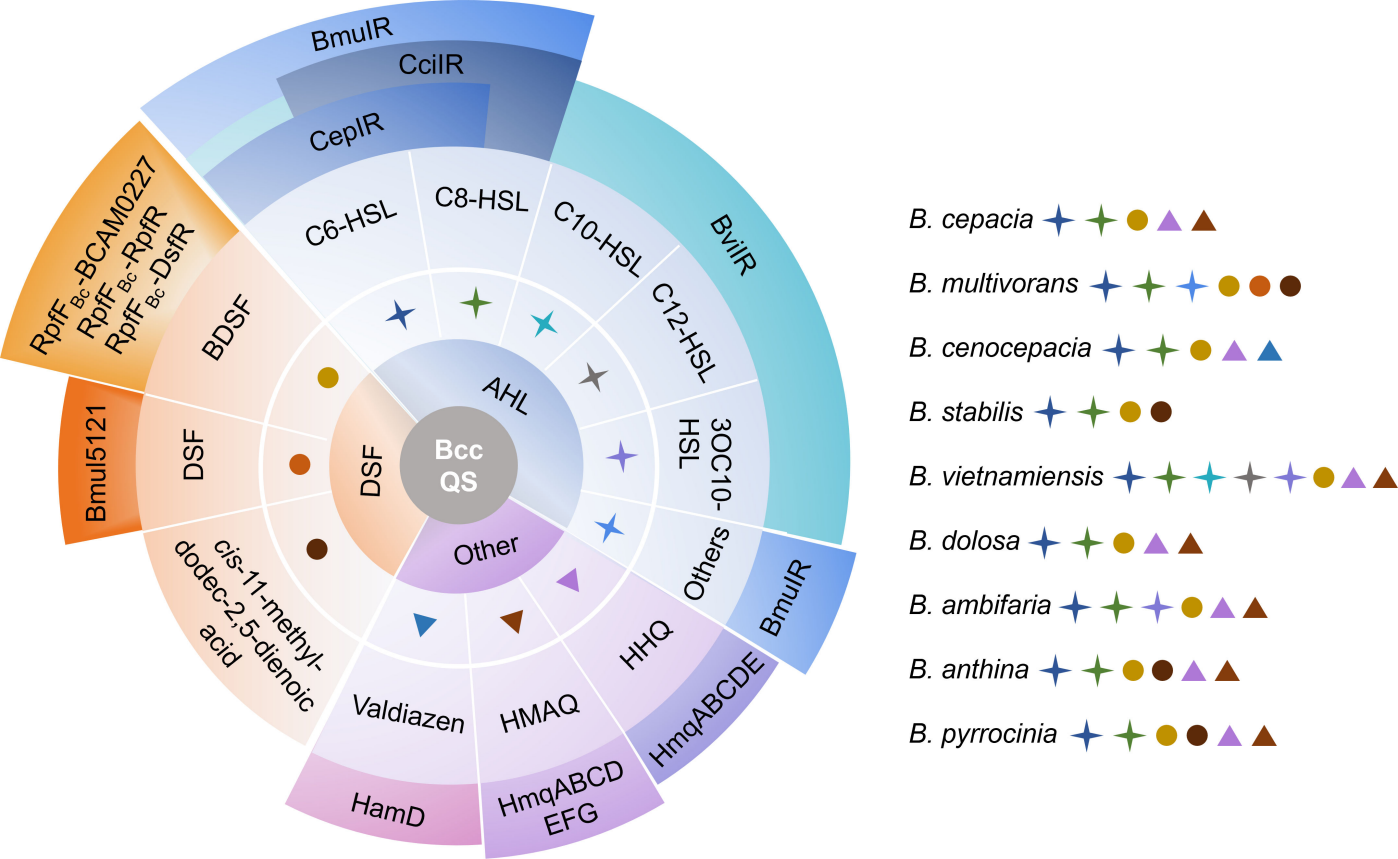
Schematic diagram of the Bcc QS. The Bcc species produce the DSF-type signals and the AHL-type signals, as well as other signals including Valdiazen, HMAQ, and HHQ. The synthases or receptors responsible for transmitting QS signals and their identified signaling pathways are also presented.

The quinolone signal HHQ (2-heptyl-4-quinolone) was first characterized in *P. aeruginosa* as both a signaling molecule and biosynthetic precursor for PQS (136). HHQ is synthesized by the *pqsABCDE* operon, with PqsA acting as the essential initiator enzyme. PQS is subsequently generated via the monooxygenase PqsH, which hydroxylates HHQ at the C3 position. Homologs of the *pqsABCDE* operon (initially named *hhqABCDE*, and later renamed *hmqABCDE*) are conserved in some strains in the Bcc, though these species exclusively produce HHQ without further conversion to PQS. (Fig. 5) (136–139). Further research has revealed that the *hmqABCDEFG* operon structurally modifies HHQ derivatives through HmqF-mediated C3 methylation and HmqG-catalyzed alkyl chain desaturation to generate HMAQs in Bcc strains such as *B. ambifaria*, *B. cepacia, B. pyrrocinia, B. vietnamiensis*, *B. anthina,* and *B. dolosa*. The *hmqABCDEFG* operon primarily produces HMAQs, with HHQ as a minor biosynthetic intermediate (140–142). Notably, early studies reported the detection of alkylquinolones, including HHQ, in *B. cenocepacia* (139). However, subsequent and more systematic research confirmed that *B. cenocepacia* generally lacks the complete *hmqABCDEFG* biosynthetic operon and does not produce its characteristic product, HMAQs (142).

HHQ/HMAQ modulates multiple bacterial phenotypes, ranging from biofilm maturation to virulence factor secretion. A fundamental distinction from *P. aeruginosa* is the absence of identified cognate receptors (e.g., PqsR) for HHQ/HMAQ in *Burkholderia*, implying that their phenotypic modulation likely occurs through receptor-independent mechanisms such as membrane interaction, enzyme allostery, or indirectly via crosstalk with established QS systems like CepIR (105, 143, 144). Functional evidence from *B. ambifaria* demonstrates that HMAQ deficiency dysregulates the CepIR system, causing aberrant AHL accumulation and consequent disruptions in biofilm formation and virulence (105). Ecologically, the *hmq* operon is enriched in plant root-associated *Burkholderia* species (e.g., *B. gladioli*) but absent in clinical isolates from people with CF, suggesting adaptive specialization for rhizosphere colonization (142). Despite unresolved signaling mechanisms, HHQ biosynthesis remains a viable therapeutic target; structure-guided inhibitors of HmqA effectively block HHQ production and attenuate virulence in pathogenic *Burkholderia*.

### Valdiazen

Jenul et al. identified a fragin biosynthesis-related gene cluster in the *B. cenocepacia* strain H111 (145). Transcriptomic profiling (RNA-Seq) revealed that specific genes within the *ham* cluster drive the production of the diazeniumdiolate-class signaling molecule valdiazen (Fig. 5). This molecule displays autoregulatory properties, not only by activating its own biosynthesis but also by coregulating fragin synthesis and modulating the expression of more than 100 downstream genes.

The valdiazen biosynthetic pathway involves a multistep enzyme-catalyzed reaction. First, the nonribosomal peptide synthetase (NRPS)-related adenylation enzyme HamD primes L-valine onto a phosphopantetheinyl carrier protein. Then, sequential hydroxylation and nitration yield nitro-functionalized intermediates. Final assembly, through stereoselective reduction and transaminase-mediated amination, results in a bioactive diazeniumdiolate structure (145).

## CONCLUSIONS AND PROSPECTS

The Bcc, a highly diverse and globally distributed pathogen, has multifaceted impacts on human health, particularly in immunocompromised populations such as people with CF and CGD (7, 53, 54). It encompasses various types, which can be broadly classified into human pathogenic bacteria, plant pathogenic bacteria, plant growth-promoting rhizobacteria, and biodegrading bacteria. In Bcc pathogens, QS functions as an integrated regulatory network comprising multiple interconnected systems. This network coordinately controls the expression of diverse genes, including key virulence determinants and pathogenicity modulators, in response to population density and environmental cues (12, 22, 79, 107, 146). Given that clinical Bcc pathogens exhibit high levels of drug resistance, conducting in-depth research and comprehensively understanding the role of QS regulation in pathogenic virulence mechanisms, its inherent patterns, as well as the classification components and regulatory networks of the QS system, hold significant theoretical importance and practical value for developing novel antimicrobial therapeutic strategies targeting Bcc infections.

QS in Bcc exhibits remarkable diversity and complexity. The identified QS signals in the Bcc species primarily include AHL-type molecules (e.g., C6-HSL, C8-HSL, C10-HSL, C12-HSL, 3OC10-HSL), DSF-family molecules (e.g., BDSF, DSF, and *cis*-11-methyl-dodec-2,5-dienoic acid), as well as HHQ, HMAQ, and Valdiazen signals (Fig. 5). Among these signals, C6-HSL, C8-HSL, and BDSF are widely distributed across Bcc species, whereas C10-HSL, C12-HSL, and 3OC10-HSL are predominantly found in *B. vietnamiensis* (78, 85, 86). The molecule *cis*-11-methyl-dodec-2,5-dienoic acid is associated primarily with *B. multivorans, B. anthina, B. stabilis,* and *B. pyrrocinia*, whereas DSF is present mainly in *B. multivorans* (17, 23). Among the intricate Bcc QS systems, LuxIR-type systems (e.g., CepIR) display highly conserved signaling pathways, are widely distributed across the Bcc species, and are structurally/functionally analogous to those in other Gram-negative bacteria. In contrast, certain QS systems exhibit species specificity, such as the BviIR system unique to *B. vietnamiensis* (12). The DSF-family signaling pathways present distinct evolutionary characteristics. Although the BDSF signal receptor Bcam0227 (113) in Bcc is conserved with the DSF receptor RpfC in *Xanthomonas campestris* (Xcc) (147), the RpfF_Bc_-RpfR (18) pathway in Bcc differs entirely from the RpfF-RpfC-RpfG pathway in Xcc. Notably, the newly identified RpfF_Bc_-DsfR (131) pathway not only diverges from the canonical Xcc pathway but also shows significant differences from the RpfF_Bc_-RpfR pathway in Bcc strain H111, indicating that the BDSF signal has evolved unique novel signal transduction mechanisms while retaining conserved features of the DSF-family QS signals. Bioinformatics analyses suggest that both the Rpf_Bc_-RpfR pathway and the RpfF_Bc_-DsfR pathway may be broadly present in other bacterial species, highlighting the potential universality of these regulatory mechanisms. The coexistence of conserved and species-specific Bcc QS systems offers dual strategies for antimicrobial development: targeting conserved pathways (e.g., LuxIR-type systems) may enable the design of broad-spectrum QS inhibitors (QSIs), whereas species-specific pathways (e.g., certain DSF family members) may serve as precision targets for tailored antimicrobial therapies.

Targeting QS as a strategy for developing novel antimicrobial agents represents a critical research direction in the field of anti-infective therapy. In recent years, researchers have made groundbreaking progress in elucidating QS regulatory mechanisms and developing QSIs. For example, suppressing bacterial virulence by targeting key QS regulatory components has inspired the development of potential innovative drugs. Oridonin was found to inhibit the expression of BDSF and AHL synthase-encoding genes by directly binding to RqpR, a regulatory factor in the two-component system RqpSR, and interacting with CepR, a transcriptional regulator in the *cep* AHL system, thereby disrupting critical *B. cenocepacia* phenotypes such as motility, biofilm formation, protease production, and virulence (26). A novel structural analog of BDSF, 14-Me-C_16:Δ2_ (*cis*-14-methylpentadec-2-enoic acid), suppresses BDSF production and impairs BDSF-regulated phenotypes in *B. cenocepacia*, including motility, biofilm formation, and virulence, without affecting the bacterial growth rate (24). PQS delivered via outer membrane vesicles from *P. aeruginosa* or liposomes attenuates *B. cenocepacia* virulence to target the LysR-type regulator ShvR, demonstrating a therapeutic effect in animal infection models (26). Emerging evidence suggests that host organisms can detect QS signals and mount adaptive responses, indicating the potential for cross-kingdom QS pathway interference to combat bacterial infections. For example, Moura-Alves et al. demonstrated through *in vitro* and *in vivo* experiments that the host aryl hydrocarbon receptor senses QS molecules secreted by *P. aeruginosa* during different infection stages, coordinating host defense mechanisms accordingly (148).

Compared with conventional antibiotics, QSIs offer distinct advantages: they interfere with bacterial pathogenic signaling rather than directly killing bacteria, thereby reducing selection pressure and delaying resistance development. QSIs exhibit enhanced specificity against biofilms and chronic infections while causing minimal disruption to the host microbiota (149). However, the current limitations of QSIs include delayed efficacy due to density-dependent mechanisms and potential dysbiosis risks from non-specific inhibition of QS systems in commensal bacteria—such as AHL-mediated signaling in gut *Bacteroides*—which necessitates pathogen-specific compound design to spare beneficial microbiota (150). Despite incomplete understanding of the signaling network, QS-targeted strategies demonstrate unique promise against multidrug-resistant Bcc pathogens, with future clinical translation requiring rigorous ecological risk assessments and optimized species-selective agents like *B. cenocepacia*-specific CepR antagonists. Owing to their low risk of resistance development and host-friendly properties, QSIs are crucial for future anti-virulence development, offering significant research value and promising clinical application prospects.

In summary, the existing research on Bcc QS has laid a theoretical foundation for the development of new strategies to prevent and treat pathogenic infections. This review of Bcc QS not only advances our understanding of bacterial pathogenesis but also paves the way for innovative therapies against these formidable pathogens. Future research must address species diversity, mechanistic specificity, and clinical translational feasibility to fully harness the potential of QS inhibition.
